# Whole-Genome Sequencing of Resistance, Virulence and Regulation Genes in Extremely Resistant Strains of *Pseudomonas aeruginosa*

**DOI:** 10.3390/medsci13010006

**Published:** 2025-01-03

**Authors:** Nerlis Pajaro-Castro, Erick Diaz-Morales, Kenia Hoyos, Cristhian Ibañez-Bersinger

**Affiliations:** 1 Medical and Pharmaceutical Sciences Group, Faculty of Health Sciences, University of Sucre, Sincelejo 700001, Sucre, Colombia; cibanezb@gmail.com; 2 Salud Social Clinic, Sincelejo 700001, Sucre, Colombia; erick76dm@yahoo.es (E.D.-M.); kmhoyosgonzalez@gmail.com (K.H.)

**Keywords:** virulence genes, regulatory genes, efflux pumps, beta-lactamases, biofilms, resistance genes, secretion systems

## Abstract

Background/Objectives: *Pseudomonas aeruginosa* is a clinically significant opportunistic pathogen, renowned for its ability to acquire and develop diverse mechanisms of antibiotic resistance. This study examines the resistance, virulence, and regulatory mechanisms in extensively drug-resistant clinical strains of *P. aeruginosa*. Methods: Antibiotic susceptibility was assessed using the Minimum Inhibitory Concentration (MIC) method, and whole-genome sequencing (WGS) was performed on the Illumina NovaSeq platform. Results: The analysis demonstrated a higher prevalence of virulence genes compared to resistance and regulatory genes. Key virulence factors identified included secretion systems, motility, adhesion, and biofilm formation. Resistance mechanisms observed comprised efflux pumps and beta-lactamases, while regulatory systems involved two-component systems, transcriptional regulators, and sigma factors. Additionally, phenotypic profiles were found to correlate with resistance genes identified through genotypic analysis. Conclusions: This study underscores the significant resistance and virulence of the clinical *P. aeruginosa* strains analyzed, highlighting the urgent need for alternative strategies to address infections caused by extensively drug-resistant bacteria.

## 1. Introduction

*Pseudomonas aeruginosa* is a Gram-negative opportunistic bacterium that has become a significant public health concern due to its association with high rates of morbidity and mortality [1]. It is a major cause of severe infections, primarily in hospital settings, including pneumonia, urinary tract infections, wound infections, and bloodstream infections [2]. In 2021, bacterial resistance was linked to an estimated 4.71 million deaths worldwide, underscoring the severity of this growing threat [3]. Treating *P. aeruginosa* infections remains particularly difficult because of the bacterium’s remarkable ability to mutate rapidly and adapt to antibiotics [2,4].

The genome of *P. aeruginosa* spans 5.5 to 7 Mbp and is packed with genes encoding transporters, transcriptional regulators, and two-component systems. These features equip the bacterium with an impressive metabolic versatility that allows it to adapt to diverse environmental conditions [5]. Its genetic diversity stems from a combination of a conserved core genome and a highly variable accessory genome, enabling inter-bacterial communication and the exchange of genetic material through horizontal gene transfer [6]. This adaptability not only supports its survival in challenging environments but also contributes to its antimicrobial resistance and virulence, enhancing its ability to cause infections and evade treatment [7].

The antimicrobial resistance mechanisms in *P. aeruginosa* can be grouped into three categories: intrinsic, acquired, and adaptive. Intrinsic resistance is primarily due to the low permeability of its outer membrane, the expression of efflux pumps, and the production of antibiotic-inactivating enzymes [8,9]. For example, the porin OprD, which facilitates the uptake of carbapenems, is often absent in resistant strains, leading to reduced susceptibility to these antibiotics [10]. Additionally, *P. aeruginosa* has twelve efflux pumps from the RND family, with four (MexAB-OprM, MexCD-OprJ, MexEF-OprN, and MexXY-OprM) playing key roles in antibiotic resistance [11]. It also produces β-lactamase enzymes through an inducible *ampC* gene, rendering β-lactam antibiotics ineffective [5].

Acquired resistance, on the other hand, occurs when the bacterium gains resistance genes through horizontal gene transfer via mobile genetic elements such as integrons, transposons, or plasmids [9]. For instance, between 2013 and 2015, Rada et al. [12] identified *P. aeruginosa* strains in Colombian hospitals that carried the *bla_VIM-2* and *bla_KPC-2* genes. These strains also exhibited mutations in quinolone resistance-determining regions (*gyrA*, *parC*, and *parE*) and in regulatory genes (*mexZ*, *nfxB*, *mexT*, and *mexR*). Notably, polymorphisms in the *ampC* gene, such as the T105A substitution, were linked to increased resistance to carbapenems and cefepime. Adaptive resistance mechanisms include biofilm formation and the development of persister cells, both of which further complicate treatment [5].

*P. aeruginosa* is also equipped with numerous virulence factors that make it a formidable pathogen. These include flagella, pili, and lipopolysaccharides (LPS) that facilitate adhesion and colonization, as well as secretion systems that deliver toxins and effectors to host cells. Proteases and other toxins contribute to tissue damage, while biofilm formation aids in bacterial communication and antibiotic resistance [7,13,14,15]. In a study conducted in Egypt, Edward et al. [16] found that biofilm formation (89.4%) was the most common virulence factor among isolates, whereas DNase activity was the least prevalent (10.6%). Similarly, Abril et al. [17] highlighted the genomic plasticity of *P. aeruginosa* ST235, which allows it to acquire, maintain, and modify foreign DNA. This ability is crucial for adapting to hostile environments, such as those with high antibiotic concentrations.

As antimicrobial resistance continues to rise and new drug development slows, timely susceptibility testing is becoming increasingly important to ensure effective treatments. However, conventional microbiological methods are slow, requiring 24 to 48 h for results due to bacterial growth times. While polymerase chain reaction (PCR)-based methods offer faster results, they may not cover the growing diversity of resistance mechanisms. In this context, genomic sequencing emerges as a promising diagnostic tool [18]. Genomic surveillance of *P. aeruginosa* is particularly valuable for identifying and monitoring resistance mechanisms and virulence genes, especially in multidrug-resistant (MDR) or extensively drug-resistant (XDR) strains with limited treatment options. Such efforts can guide infection control strategies and promote the rational use of antibiotics in healthcare settings. Understanding these dynamics is essential to predict resistance trends, optimize therapeutic approaches, and curb the spread of resistant strains. This study aims to identify the key resistance, virulence, and regulatory mechanisms in XDR *Pseudomonas aeruginosa* strains isolated from a clinic in Sincelejo, Sucre.

## 2. Materials and Methods

The study design was as follows: This retrospective experimental study aimed to analyze resistance mechanisms in extensively drug-resistant (*XDR*) *Pseudomonas aeruginosa* strains isolated from Clínica Salud Social in Sincelejo, Sucre, Colombia. Clinical samples were collected from patients as part of routine diagnostics between January 2022 and March 2024. The multidrug-resistant strains, identified as being of public health concern, were cryopreserved in skim milk media at −20 °C. For this study, seven strains were randomly selected from the preserved collection. To identify *Pseudomonas aeruginosa* strains, antimicrobial resistance profiles recorded in WHONET software version 5.6 during the same time period were reviewed. XDR strains were then randomly selected for further analysis. A review of patient records associated with these strains revealed that 51% of the patients had not received prior antibiotic treatment, while 49% had. Among those who had received treatment, 75% were administered beta-lactam antibiotics, and 25% were treated with carbapenems.

Phenotypic testing was conducted in the following way: The strains were thawed at room temperature for 30 min, and sterile calibrated loops were used to inoculate the microorganisms onto enriched (chocolate agar) and differential (MacConkey agar) media using the streaking method to confirm strain purity and viability. Cultures were incubated aerobically at 37 °C for 24 h, and Gram staining was performed for microscopic analysis.

Antibiotic susceptibility testing was conducted using the Minimum Inhibitory Concentration (MIC) method. Strain suspensions were adjusted to a turbidity range of 0.5–0.64 McFarland using the DensiCHECK instrument (BioMérieux, Durham, NC, USA). Antimicrobial susceptibility was evaluated with AST-93 and AST-403 cards on the VITEK^®^ 2 system. The antibiotics tested included β-lactam/β-lactamase inhibitor combinations (piperacillin/tazobactam, ceftazidime/avibactam), cephalosporins (cefazolin, ceftazidime, cefepime), monobactams (aztreonam), carbapenems (meropenem), aminoglycosides (amikacin), and fluoroquinolones (ciprofloxacin). The results were interpreted based on MIC (μg/mL) cutoff values provided in the CLSI M100 guidelines (CLSI, 2024). The strains were classified as susceptible (S), intermediate (I), or resistant (R) according to these criteria. Microorganisms were designated as multidrug-resistant (MDR) if they exhibited resistance to at least one antibiotic in three or more classes, and as extensively drug-resistant (XDR) if resistant to at least one antibiotic in six or more evaluated classes. Carbapenemase production was assessed qualitatively using the Rapidec Carba NP test, a colorimetric assay designed for the direct detection of carbapenem hydrolysis. *Pseudomonas aeruginosa* ATCC 27853 was included as a quality-control strain.

Genotypic testing was conducted as follows: The isolates were sent to the National Center for Genomic Sequencing (CNSG) at the University of Antioquia for whole-genome sequencing. Genomic DNA (gDNA) was extracted using the Qiagen DNeasy PowerLyzer PowerSoil Kit, following the manufacturer’s protocol. The extracted gDNA was quantified by measuring light absorption at 260 nm using the NanoDrop™ 2000 spectrophotometer (Thermo Scientific™, Wilmington, DE, USA) and was stored at −20 °C for subsequent sequencing. Sequencing was performed using TruSeq Nano DNA (350) libraries on the Illumina NovaSeq platform, generating 150-base paired-end reads.

Analysis of resistance genes and virulence factors was carried out in the following manner: Antimicrobial resistance genes were identified using the Resistance Gene Identifier (RGI) algorithm, based on the Comprehensive Antibiotic Resistance Database (CARD). Virulence factors were identified using the Virulence Factor Database (VFDB). Additionally, plasmid sequences were detected in the assembled contigs using PlasmidFinder, which assessed the presence of mobile genetic elements implicated in the horizontal transfer of resistance and virulence genes. These analyses provided a detailed overview of the genetic mechanisms underlying antimicrobial resistance and pathogenicity in the analyzed strains.

Gene filtering and analysis based on identity and functional classification were carried out in the following way: Data filtering was conducted using a maximum identity threshold of 90% to identify genes with a relatively high similarity. The analysis was performed in Python version 3.10.14, leveraging the pandas library for data processing and organization. The results were systematically compiled into an Excel file titled genes_90.xlsx, which included multiple worksheets: a general sheet listing all identified genes, individual sheets for each strain analyzed, and a summary sheet containing comparative statistics across strains. This structure facilitated detailed analysis of specific genes and cross-strain comparisons. A systematic evaluation of resistance and virulence genes was also performed to classify them by their primary functions and mechanisms of action. Data are presented as percentages and mean ± standard deviation, providing quantitative insights into the findings.

Ethical implications were as follows: This research adheres to the ethical guidelines established by the Colombian Ministry of Social Protection, as outlined in Resolution 8430 of 4 October 1993 [19] and Resolution 2378 of 2008 [20], as well as the Declaration of Helsinki [21]. The study is classified as risk-free, as it relies on retrospective documentary research methods and techniques without any intentional intervention or modification of the biological, physiological, psychological, or social variables of the study participants. All information and cell cultures used in the study were provided by Clínica Salud Social.

## 3. Results

The bacterial strains analyzed in this study were isolated from hospitalized patients, 86% of whom were male and 14% female, with the majority (86%) being over 50 years of age. Prior antibiotic treatment was documented in 49% of the patients, predominantly involving beta-lactams (75%) and carbapenems (25%). All patients had an indwelling intravenous catheter, and 28.6% also had a urinary catheter. Most infections (71.4%) were detected upon hospital admission through positive urine cultures, while the remaining cases were identified during the course of hospitalization. Notably, 57.1% of the patients required extended hospital stays lasting more than two weeks. All strains demonstrated resistance to carbapenems.

The results obtained in the phenotypic evaluation (Table 1) of the seven strains studied show that they are resistant to all classes of antibiotics evaluated, and, therefore, these strains are extremely drug-resistant.

**Figure 1:**
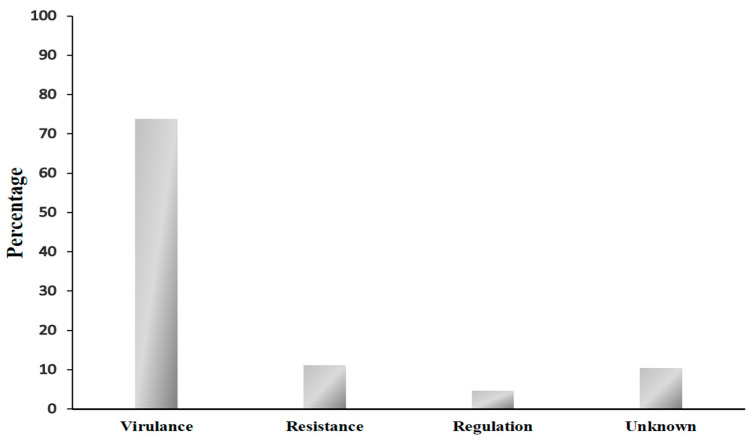
Classification of genes found in *P. aeruginosa* strains.

The analysis of resistance and virulence genes based on the CARD and VFDB databases identified an average of 432 ± 11 genes. After filtering for genes with greater than 90% identity, the average number of genes decreased to 415 ± 8, with strain 637,345 possessing the fewest genes (402) and strain 629,590 the most (425). A total of 367 genes were common to all bacterial strains and were categorized according to their resistance mechanisms and phenotypic profiles. Figure 1 illustrates the classification of these genes into virulence, resistance, regulatory, and unknown categories, with virulence genes being predominantly identified (271). Resistance genes accounted for 41, regulatory genes for 17, and unknown genes for 38.

**Figure 2:**
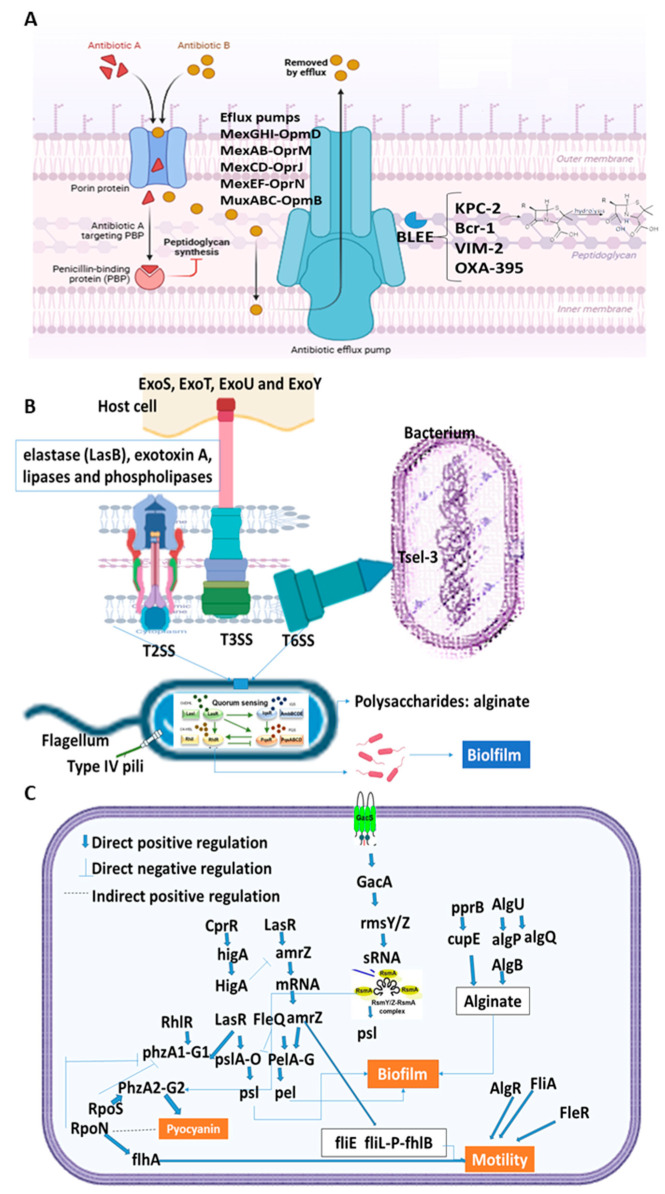
Main resistance mechanisms (**A**), virulence (**B**), and regulation (**C**) detected in *P. aeruginosa*. Part a shows two resistance mechanisms, the efflux pumps which are responsible for expelling antimicrobial drugs, and enzymes that hydrolyze antibiotics, which are four ESBLs, KPC-2 and bcr-1 (Class A), VIM-2 (Class B), and OXA-395 (Class D). Part b shows the virulence mechanisms, secretion systems (T6SS, T3SS, and T2SS), motility (flagellar system), adhesion (type IV pili, cup fimbriae), and biofilm formation. Part c highlights the regulatory genes that participate in the activation of motility, biofilm formation, and pyocyanin in which two-component systems, transcriptional regulators, and sigma factors participate.

Additionally, the types of genes in each category were analyzed to identify the highest percentage of specific gene types and to establish mechanisms of virulence, resistance, and regulation (Table 2). The main resistance, virulence, and regulatory mechanisms identified in the studied strains are shown in Figure 2. Enzymes that inactivate antibiotics and efflux pumps were the primary resistance mechanisms, while secretion systems, motility, adhesion, and biofilm formation were the main virulence mechanisms. For regulation, two-component systems, transcriptional regulators, and sigma factors were identified.

Additionally, a detailed analysis of unique genes revealed that only strains 637,345 and 547,256 harbored these genes, as outlined in Table S1. Among these, nine were identified as virulence-related, seven were associated with resistance, and two were regulatory genes.

Finally, considering the phenotypic resistance profiles of each strain, the presence of resistance genes corresponding to the tested antimicrobials was analyzed (Table S2). This analysis confirmed the association between the identified resistance genes and the observed resistance patterns in the strains. Furthermore, additional resistance genes were detected, including those conferring resistance to other classes of antimicrobials and antiseptics, underscoring the broad adaptability of these strains.

## 4. Discussion

In this study, seven *Pseudomonas aeruginosa* strains were analyzed to determine their resistance profiles across various antibiotic classes and their genetic characterization in terms of resistance, virulence, and regulatory mechanisms. Phenotypic analyses revealed extensive resistance across all tested antibiotic classes, including β-lactams, aminoglycosides, fluoroquinolones, and glycylcyclines (Table 1), posing a substantial clinical challenge. This observation aligns with previous reports describing *P. aeruginosa* as a prominent opportunistic pathogen, notable for its intrinsic resistance and capacity to acquire additional resistance mechanisms, making it a significant threat in healthcare-associated infections [5,22]. Abb et al. (2020) reported that 96% of *P. aeruginosa* isolates were multidrug-resistant (MDR), while 87% exhibited extensive drug resistance (XDR) [23]. Similarly, a 2022 study in Bogotá utilizing whole-genome sequencing identified 95 distinct resistance-associated genes in clinical *P. aeruginosa* isolates. Of these, 51.6% were linked to efflux pumps, 35.2% to enzymatic antibiotic inactivation, 9.9% to target site alterations, 2.2% to reduced porin production, and 1.1% to porin absence [24]. Notably, the MexAB-OprM efflux system was detected in all isolates, with the genes mexA, mexB, and oprM consistently present.

Genomic analysis revealed a high proportion of virulence-associated genes (73.8%) in the studied strains (Figure 1), underscoring a robust genetic arsenal that facilitates infection establishment and immune evasion. Additionally, 11.2% of the identified genes were associated with antibiotic resistance, potentially explaining the observed phenotypic profiles, while 4.6% were regulatory genes, highlighting the importance of genetic regulation in modulating both virulence and resistance. Notably, 10.4% of the genes remained uncharacterized, potentially representing novel functions that may contribute to bacterial adaptability.

The survival of *P. aeruginosa* in the presence of antimicrobials is supported by intrinsic, acquired, and adaptive resistance mechanisms. Intrinsic mechanisms include the bacterium’s low outer membrane permeability and efflux pumps that actively expel antibiotics. Acquired resistance involves specific genes, such as blaOXA and blaGES, conferring resistance to β-lactams, as well as genes encoding enzymatic modifications affecting other antibiotic classes. Adaptive mechanisms, driven by selective pressures in hospital environments and prolonged antibacterial therapies, promote changes in efflux pump expression and quorum-sensing regulators, enhancing both virulence and persistence. This study demonstrates that these highly resistant clinical strains employ a combination of these mechanisms, significantly complicating infection treatment.

These findings are consistent with those of Aroca et al. (2024) [25], who identified 239 virulence-associated genes in *P. aeruginosa* isolates, 62.3% of which (149 genes) were shared between clinical and environmental isolates. Among these shared genes, 85 were associated with adhesion and motility, 76 with type II, III, and VI secretion systems, and 46 with quorum-sensing pathways (lasI/lasR and rhlI/rhlR). Aroca et al. [25] also identified 135 resistance-associated genes, 72.6% (98 genes) of which were shared across analyzed genomes, encompassing mechanisms such as efflux pumps, membrane permeability alterations, antibiotic inactivation, and target modification [25]. Similarly, Edward et al. [16] highlighted that high virulence, combined with resistance to multiple antimicrobial agents, likely exacerbates morbidity and mortality in *P. aeruginosa* infections.

Genomic characterization further revealed a high prevalence of virulence-associated genes across all analyzed strains, including those linked to secretion systems, motility, adhesion, and biofilm formation (Table 2). Notably, secretion systems types II, III, and VI (T2SS, T3SS, and T6SS) were well-represented, with 83 genes identified. These systems play a pivotal role in pathogenicity by mediating the translocation of toxic effectors into the extracellular environment or directly into host cells, facilitating immune evasion, tissue invasion, and the disruption of cellular functions. Moreover, they contribute to bacterial competition by delivering toxic proteins into competing bacterial cells, leading to cell wall degradation or membrane disruption, which can weaken or eliminate rival bacteria [26,27,28,29].

Motility-related genes, primarily associated with the flagellar system and chemotaxis, indicate that these strains are highly motile—an essential characteristic for surface colonization and dissemination within hospital environments. Adhesion systems, such as type IV pili and cup fimbriae, are critical for adherence, colonization, and biofilm formation [26,29]. Bacterial biofilms consist of structured microbial communities embedded within an extracellular polysaccharide matrix (EPS) produced and secreted by the bacteria, allowing surface attachment [29,30]. Abdulhaq et al. [31] reported that MDR strains commonly form biofilms, with the pslA gene detected in all biofilm-forming isolates. Another study found that MDR *P. aeruginosa* strains exhibited combined virulence patterns, including adhesion and cytotoxicity, with key virulence genes such as aprA, exoU, exoS, lasB, algD, and toxA detected [32].

The detection of high levels of resistance to beta-lactams, including carbapenems such as meropenem, is strongly associated with the presence of genes like *KPC-2*, *VIM-2*, and *OXA-395*, which encode β-lactamases capable of inactivating a broad spectrum of beta-lactam antibiotics. Carbapenem resistance in *P. aeruginosa* is particularly alarming, as these antibiotics are often considered the last line of defense for severe infections. Strains of *P. aeruginosa* have been reported to harbor various extended-spectrum β-lactamases (*OXA-14*, *OXA-19*, *OXA-35*, *GES-9*, and *PER-1*), carbapenemases (*GES-5*, *IMP-8*, and *VIM-2*), or combinations of both (e.g., *VIM-2*/*OXA-35* and *VIM-4*/*SHV-2a*). These strains, often associated with epidemic clones, such as ST111, ST175, CC235, ST244, ST348, and ST654, exhibit resistance to nearly all tested antibiotics, with the exception of colistin [33]. A recent study highlighted that resistome analysis via whole-genome sequencing (WGS) of respiratory isolates revealed a significant diversity of horizontally acquired resistance genes and mutations, frequently linked to major high-risk clones with global dissemination. ST235, for instance, was dominant and characterized by multiple horizontally acquired β-lactamases and mutation-driven resistance mechanisms. Even isolates from a single center displayed multiple independent ST235 lineages, emphasizing the broad dispersal of this high-risk clone. Notably, co-production of class B carbapenemase *NDM-1* and class A carbapenemase *GES-5* in ST654 isolates, previously unreported, was observed [34]. Resistance to combinations like ceftazidime/avibactam and piperacillin/tazobactam suggests the operation of highly efficient resistance mechanisms, such as the synergistic effects of efflux pumps (e.g., MexAB-OprM and MexEF-OprN) and β-lactamase enzymes, further narrowing therapeutic options. Efflux pump-related resistance genes were prevalent in the analyzed strains, with 33 genes identified, primarily from families such as *MexGHI-OpmD* and *MexAB-OprM*. These pumps are known for their broad substrate specificity, which includes antibiotics and toxic compounds. Additionally, resistance genes linked to lipopolysaccharide (LPS) modifications and two-component regulatory systems, such as *arnA*, *ParS*, and *basS*, indicate polymyxin resistance, further complicating treatment strategies [30,35]. Consistent with these findings, Almaghrabi et al. [36] reported that all examined *P. aeruginosa* isolates carried genes encoding the efflux pumps MexAB-OprM, MexCD-OprJ, MexEF-OprN, MexGHI-OpmD, MexJK-OprM, MexVW-OprM, MexPQ-OpmE, MuxABC-OpmB, MexMN-OprM, TriABC-OpmH, EmrE, and PmpM, many of which were also detected in our study.

Furthermore, the regulatory genes identified in this study predominantly include two-component systems, transcriptional regulators, and sigma factors, which collectively enable *P. aeruginosa* to adapt to environmental changes and stress. Systems like GacS/GacA and CprRS modulate the expression of virulence and resistance factors under stress conditions, while sigma factors RpoN and RpoS play key roles in nutrient-limited environments and other stress responses, which are common in chronic infections [26,37]. These regulatory mechanisms contribute significantly to the pathogen’s ability to survive and thrive in adverse settings, such as hospital environments.

Additionally, unique genes were identified in certain strains (Table S1), notably in strains 637,345 and 547,256, which were associated with both resistance and virulence. For instance, resistance genes such as *mphE* and *APH*(3′)-VI confer resistance to macrolides and aminoglycosides, respectively. Meanwhile, virulence genes like *pldA* and *exoU*, linked to secretion systems and toxin production, may enhance the pathogenic potential of these strains. These findings underscore the genetic diversity of *P. aeruginosa* and its capacity to adapt its resistance and virulence repertoire to specific environmental or clinical conditions. This adaptability highlights the critical need for personalized therapeutic strategies and ongoing surveillance of resistance mechanisms in clinical settings [26].

The results of this study align with prior research on the mechanisms underlying *Pseudomonas aeruginosa* resistance to multiple antibiotic classes, particularly highlighting the high prevalence of resistance among MDR and XDR strains [34]. The identification of resistance genes in the studied isolates corroborates their phenotypic resistance profiles, as presented in Table S2. Torrens et al. (2022) [34] documented a significant prevalence (>20%) of resistance to novel β-lactam/β-lactamase inhibitor combinations, with up to 83% of XDR isolates exhibiting resistance to ceftolozane/tazobactam and/or ceftazidime/avibactam. Similarly, our study identified resistance genes associated with these antimicrobials. The overexpression of efflux pump genes, such as *mexY* and *mexX*, observed in this study is consistent with findings by Saeli et al. [38], who reported a high prevalence of these genes in aminoglycoside-resistant strains and their contribution to biofilm formation, enhancing bacterial persistence in clinical environments. Zahedi et al. [39] also highlighted the frequent expression of the MexAB-OprM and MexCD-OprJ efflux pump systems in strains resistant to ticarcillin and ciprofloxacin, which aligns with our observation of RND-type efflux pump overexpression in beta-lactam- and fluoroquinolone-resistant strains. Colistin resistance was detected in the analyzed strains, consistent with findings by Abd El-Baky et al. [23], who reported a high prevalence of the *mcr*-*1* gene in colistin-resistant strains. Our study identified *mcr*-*2*, further supporting the conclusion that colistin resistance in *P. aeruginosa* is primarily mediated by *mcr* genes and lipid A modifications involving *arnA* and regulatory systems such as ParS and BasS. MDR efflux pumps, which are highly conserved across bacterial species, contribute to intrinsic and acquired resistance, while also playing roles in virulence, quorum sensing, and the detoxification of metabolic intermediates and toxic compounds, including heavy metals and antimicrobials. Notably, efflux gene expression is a more reliable predictor of efflux-mediated resistance than the mere presence of efflux pump genes [40]. Aminoglycoside resistance in our strains aligns with the research of El-Far et al. [41], which reported high frequencies of resistance genes like *rmtB*, *armA*, and *aac*(*6*′)-*Ib*, reflecting their rapid dissemination in clinical environments. The elevated expression of *phoP* genes was also observed, reinforcing their role in aminoglycoside resistance and contribution to the XDR phenotype, as noted in previous studies. Ahmed [42] similarly reported that strains with higher antibiotic resistance exhibited greater acquisition of resistance genes. MDR *P. aeruginosa* isolates in this study harbored resistance genes targeting aminoglycosides, β-lactams, fluoroquinolones, sulfonamides, phenicols, and fosfomycin. Additionally, genes conferring resistance to quaternary ammonium compounds and triclosan—commonly used clinical antiseptics—were identified, underscoring their potential impact on infection control. Our findings highlight the importance of integrating phenotypic resistance testing with genotypic analysis to enhance the precision of antimicrobial management and clinical decision-making. The observed correlation between phenotypic and genotypic resistance reinforces the necessity of continuous surveillance and genomic characterization of high-risk clones, as advocated by recent studies [43,44]. Effective antibiotic stewardship and accurate diagnostics are critical quality benchmarks for patient care [45]. We recommend the active monitoring of antimicrobial resistance and virulence determinants to deepen our understanding of regulatory mechanisms in *P. aeruginosa*.

Limitations and suggestions for future work are as follows: One of the main limitations of this study was the small number of sequenced strains, limited to seven. This number restricts the generalizability of the findings to the broader population under study, highlighting the need for a larger-scale genetic surveillance study. Furthermore, no previous studies of this kind have been conducted in the Sucre department, making our research a crucial first step in understanding the resistance and virulence of *Pseudomonas aeruginosa* in the region. Future studies should focus on systematic and comprehensive monitoring of these strains, including a larger number of clinical samples, to strengthen infection control strategies in the hospital setting.

## 5. Conclusions

This study underscores the pronounced resistance and virulence of the analyzed *Pseudomonas aeruginosa* strains, along with their intricate regulatory mechanisms, highlighting the urgent need for alternative strategies to combat infections caused by extensively drug-resistant bacteria. The dynamic interplay between resistance and virulence mechanisms, reinforced by a robust regulatory network, demonstrates the exceptional adaptability of this pathogen, facilitating its persistence in clinical environments and presenting a substantial challenge to hospital infection control efforts.

## Figures and Tables

**Table 1:** Resistance pattern of the strains evaluated.

Strain	Class	Antibiotic	MIC	Interpretation
544,871 572,897 629,590 635,020 637,345 645,441 547,256 ^1^	Beta-lactam/betalactamase inhibitor	Piperacillin/Tazobactam	>=128	R
		Ceftazidime/Avibactam	>=16	R
	Cephalosporins	Cefazolin	>=64	R
		Ceftazidime	>=64	R
		Cefepime	>=64	R
	Monobactam	Aztreonam	>=64	R
	Carbapenems	Meropenem	>=16	R
	Aminoglycosides	Amikacin	>=64	R
	Fluoroquinolones	Ciprofloxacin	>=4, 2 ^1^	R, I ^1^
	Glycylcyclines	Tigecycline	>=8	R

^1^ Strain 547,256 showed intermediate susceptibility to ciprofloxacin.

**Table 2:** Classification of virulence, resistance, and regulatory genes found in *P. aeruginosa* strains.

Virulence Genes					
Subcategory	Number of Genes	Group/System	Number of Genes	% Virulence	% of Total
Secretion Systems	85	T6SS	39	31.37%	23.16%
		T3SS	33		
		T2SS	11		
		Sec	1		
		T2SS/T4P	1		
Motility	49	Flagellar system	42	18.08%	13.35%
		Chemotaxis	7		
Accession	43	type IVPili	24	15.87%	11.72%
		Fimbrias Cup	10		
		Tad System	9		
Biofilm	32	Alginate	23	11.81%	8.72%
		Amyloid formation	5		
		Rhamnolipids	3		
		Amyloid fibers	1		
Siderophores	17	Pyoverdine	17	6.27%	4.63%
Toxins	7	Phospholipases	3	2.58%	1.91%
		T3SS	2		
		T6SS	1		
		Exotoxin A	1		
Quorum Sensing	5	Las System	2	1.85%	1.36%
		Rhl system	2		
		Signaling	1		
Other mechanisms	33	Biosynthesis	8	12.18%	8.99%
		Has system	4		
		Phenazines	3		
		HCN	2		
		Protease	2		
		Elastases	2		
		T3SS	2		
		T6SS	2		
		Others	8		
Total virulence	271		271	100%	73.8%
Resistance genes					
Subcategory	Number of Genes	Group/System	Number of Genes	% resistance	% of total
Efflux pumps	33	MexGHI-OpmD	4	80.5%	8.99%
		MexAB-OprM	3		
		MexCD-OprJ	3		
		MexEF-OprN	3		
		MuxABC-OpmB	3		
		Other Mex systems	18		
β-lactamases	4	Oxacillinases	1	9.76%	1.09%
		Class B	1		
		Carbapenemases	1		
		Metallo-β-lactamases	1		
Modifying enzymes	2	Aminoglycosides	1	4.88%	0.54%
		Chloramphenicol	1		
Other mechanisms	2	LPS modification	1	4.88%	0.54%
		Resistance to sulfonamides	1		
Total resistance	41		42	100.0%	11.2%
Regulatory genes					
Subcategory	Number of Genes	Group/System	Number of Genes	% of regulators	% of total
Two-component systems	8	GacS/GacA	3	47.1%	2.18%
		CprRS	2		
		ParRS	1		
		Cpx	1		
		BasRS/PmrAB	1		
Transcriptional regulators	5	Two-component system	2	29.41%	1.36%
		AMPc	1		
		Catabolic repressor	1		
		MexR	1		
Sigma factors	2	RpoN	1	11.76%	0.54%
		RpoS	1		
Post-transcriptional regulators	1	RsmA	1	6%	0.3%
Metabolism	1	Carbon control	1	6%	0.3%
Total	17		17	100.0%	4.6%

## Data Availability

Non-digital data supporting this study are curated at the Salud Social clinic in Sincelejo-Sucre-Colombia.

## Supplementary Materials

The following supporting information can be downloaded at: https://www.mdpi.com/article/10.3390/medsci13010006/s1: Table S1: Unique genes identified in the strains studied; Table S2: Phenotype analysis with respect to the genotype of *Pseudomonas aeruginosa* strains.
