# Supplementary material for: Whole-Genome Sequencing of Resistance, Virulence and Regulation Genes in Extremely Resistant Strains of Pseudomonas aeruginosa
Source: Med Sci (Basel). 2025 Jan 3;13(1):6. doi: 10.3390/medsci13010006 (PMC11755660; doi:10.3390/medsci13010006)
Supplement: Supplementary file 1 [file medsci-13-00006-s001.zip › medsci-3333655-supplementary.pdf]

Supplementary materials

Article

Whole genome sequencing of resistance, virulence and regulation genes in extremely resistant strains of *Pseudomonas aeruginosa*

Nerlis Pajaro-Castro [1]*, Erick Diaz-Morales [2], Kenia Hoyos [3] and Cristhian Ibañez-Bersinger [4]

[1] Universidad de Sucre. Medical and Pharmaceutical Sciences Group. Postdoctoral Fellowships. Salud Social Clinic; nerlis.pajaro@unisucre.edu.co
[2] Salud Social Clinic. Sucre, Colombia; erick76dm@yahoo.es
[3] Salud Social Clinic. Sucre, Colombia; kmhoyosgonzalez@gmail
[4] Medical and Pharmaceutical Sciences Group. Universidad de Sucre; cibanezb@gmail.com
* Correspondence: nerlis.pajaro@unisucre.edu.co

Table S1. Unique genes identified in the strains studied.

Strain	Gene	Category	Subcategory	Group/System	Function	Antibiotic Resistance
637345	mphE	Resistance	Modifying enzymes	Macrolides	Macrolide phosphorylation	Macrolides (Erythromycin, Azithromycin)
	APH(3')-VI			Aminoglycosides	Phosphotransferase	Aminoglycosides (Kanamycin, Amikacin)
	msrE		Efflux pumps	MSR	ABC efflux pump	Macrolides, Streptogramins
547256	pldA/tle5a	Virulence	Toxins	T6SS	Phospholipase D	—
	exoU			T3SS	Phospholi	—

					pase A2	
	rhsP2			T6SS	Rhs toxin	—
	pvdIV		Siderophores	Pioverdina	Nonribosomal peptide synthase	—
	pvdJ					
	cupC1		Accession	Fimbrias Cup	Fimbrial subunit	—
	pilA		Accession	Type IV Pili	Main sink	—
	spcU		Secretion	T3SS	Chaperone	—
	phzG2		Secondary metabolites	Phenazines	Biosynthesis of phenazines	—
	FosA	Resistance	Modifying enzymes	Fosfomycin	Metallothioltransferase	Fosfomycin
	arr-2			Rifampicin	ADP-ribosyltransferase	Rifampicin
	PDC-385		β-lactamases	Class C	Cephalosporinase	Cephalosporins, Penicillins

	dfrA22		Resistance to trimethoprim	DHFR	Dihydrofolate reductase	Trimethoprim
	PA4541<br>PLES_RS09940	Unknown	—	—	Hypothetical protein	—

Table S2. Phenotype analysis with respect to the genotype of *Pseudomonas aeruginosa* strains.

Class	Antibiotic	Related gene
Beta-lactam/betalactamase inhibitor	Piperacillin/Tazobactam	mexY, mexX, APH(3')-IIb, OpmH, MexB, mexM, OprM (Resistance:Efflux pump), OXA-395, bcr-1 (Resistance: Beta-lactamases), ArmR (Regulation)
	Ceftazidime/Avibactam	
Cephalosporins	Cefazolin	KPC-2, VIM-2 (Resistance: Beta-lactamases), MexB, OprJ, MexD, opmE, mexN (Resistance:Efflux pump)
	Ceftazidime	
	Cefepime	
Monobactam	Aztreonam	MuxC, MuxA (Virulence: Efflux pump), OpmB, MuxB (Resistance:Efflux pump)
Carbapenems	Meropenem	MexB, opmE, mexN (Resistance:Efflux pump)
Aminoglycosides	Amikacin	mexY, mexX, APH(3')-IIb, opmE, mexQ, mexN, mexP (Resistance:Efflux pump)
	Gentamicin	
Fluoroquinolones	Ciprofloxacin	MexA, MexE, OprN, MexW, MexV, MexC, MexF, MexI, MexB, MexG, mexM, OprJ, MexH, OprM, MexD, opmE, mexQ, mexN, mexP, MexL (Resistance:Efflux pump), ArmR (Regulation)
Glycylcyclines	Tigecycline	MexX, mexY, OprM (Resistance:Efflux pump)
Quaternary compounds	Benzalkonium chloride	emrE (Resistance:Efflux pump)
nhibitor of bacterial protein synthesis	Chloramphenicol	catB7, MexI, MexB, OprM, mexQ, mexP, MexL (Resistance:Efflux pump)

Macrolides	Erythromycin	MexB, MexG, MexI, OprJ , MexH, OpmB, MexD, MuxB, OprM, mexQ, mexP, MexL, MexK, MexJ (Resistencia:Bomba de eflujo), MuxC, MuxA (Virulence: Efflux pump), ArmR (Regulation)
Polymyxins	Colistin	arnA (Resistance: Lipid A modification), ParS, basS (Resistance: Two-component systems)
Sulfonamides	Sulfamethoxazole, Sulfadiazine, Sulfisoxazole	sul1 (Resistance: Sulfonamines)
Antiseptic	Triclosan	TriB, TriA, TriC (Resistance:Efflux pump)
